# A Deficiency of Ceramide Biosynthesis Causes Cerebellar Purkinje Cell Neurodegeneration and Lipofuscin Accumulation

**DOI:** 10.1371/journal.pgen.1002063

**Published:** 2011-05-19

**Authors:** Lihong Zhao, Stefka D. Spassieva, Thomas J. Jucius, Leonard D. Shultz, H. Elizabeth Shick, Wendy B. Macklin, Yusuf A. Hannun, Lina M. Obeid, Susan L. Ackerman

**Affiliations:** 1 The Jackson Laboratory, Bar Harbor, Maine, United States of America; 2 Howard Hughes Medical Institute, Chevy Chase, Maryland, United States of America; 3 Department of Medicine, Medical University of South Carolina, Charleston, South Carolina, United States of America; 4 Department of Neuroscience, Cleveland Clinic Foundation, Cleveland, Ohio, United States of America; 5 Department of Biochemistry and Molecular Biology, Medical University of South Carolina, Charleston, South Carolina, United States of America; University of Minnesota, United States of America

## Abstract

Sphingolipids, lipids with a common sphingoid base (also termed long chain base) backbone, play essential cellular structural and signaling functions. Alterations of sphingolipid levels have been implicated in many diseases, including neurodegenerative disorders. However, it remains largely unclear whether sphingolipid changes in these diseases are pathological events or homeostatic responses. Furthermore, how changes in sphingolipid homeostasis shape the progression of aging and neurodegeneration remains to be clarified. We identified two mouse strains, flincher (*fln*) and toppler (*to*), with spontaneous recessive mutations that cause cerebellar ataxia and Purkinje cell degeneration. Positional cloning demonstrated that these mutations reside in the *Lass1* gene. *Lass1* encodes (dihydro)ceramide synthase 1 (CerS1), which is highly expressed in neurons. Both *fln* and *to* mutations caused complete loss of CerS1 catalytic activity, which resulted in a reduction in sphingolipid biosynthesis in the brain and dramatic changes in steady-state levels of sphingolipids and sphingoid bases. In addition to Purkinje cell death, deficiency of CerS1 function also induced accumulation of lipofuscin with ubiquitylated proteins in many brain regions. Our results demonstrate clearly that ceramide biosynthesis deficiency can cause neurodegeneration and suggest a novel mechanism of lipofuscin formation, a common phenomenon that occurs during normal aging and in some neurodegenerative diseases.

## Author Summary

Lipids play many essential cellular roles as structural components of biological membranes or signaling molecules. Alterations of lipids have been observed in the brains of patients with neurodegenerative diseases. However, whether these changes can cause neurodegeneration or otherwise influence the pathology of these diseases is unclear. We identified mouse mutations in a gene encoding a neuronally expressed enzyme that generates ceramide, the basic structural component of many lipids known as sphingolipids. These mutations result in progressive ataxia and loss of cerebellar Purkinje cells. In addition, many neurons in these mutant mice harbor lipofuscin, a storage material containing both membranes and proteins, that is present in aging brains and in brains of patients with neurodegenerative disorders, suggesting that both membrane and protein homeostasis are impaired in mutant neurons. This study directly demonstrates that disruption of sphingolipid biosynthesis can lead to selective neuron death and the abnormal accumulation of lipofuscin, and it underscores the need for further study of the roles of lipids in neurodegenerative disorders.

## Introduction

A hallmark of aging and many neurodegenerative disorders is the neuronal accumulation of storage materials. These deposits include lipofuscin that contain undigested membranes and defective proteins [1], and/or membrane-free aggregates of misfolded proteins [2]. While the pathological roles of these accrued substances are unclear and may vary between diseases, their sequestration may protect neurons from those components that are otherwise highly toxic in soluble forms [3]. However, evidence also suggests that insoluble storage materials are inherently toxic, and in some circumstances these materials may lead to the inhibition of proteasomal and lysosomal functions, which in turn accelerates their further deposition [4].

In addition to the accumulation of storage materials and impaired protein degradation capacity, changes in cellular homeostasis, including alterations in both simple and complex sphingolipid composition, also occur in the brains of patients with neurodegenerative diseases and in the aging brain [5], [6]. These highly diverse lipids play important structural and signaling functions in the cell, and mediate cell-cell interaction [7], [8]. Increases in levels of specific species of ceramide, the simplest sphingolipid, have been found in the brains of Alzheimer's disease (AD) patients and a mouse model of AD, and correlate with disease severity [6], [9], [10]. Similarly, long-chain and very long-chain ceramide species are increased in the brains of HIV-associated dementia patients [11]. Changes in several sphingolipid classes have been observed in the brains of patients with progressive epilepsy with mental retardation (EPMR), a form of neuronal ceroid lipofuscinosis (CLN8) [12]. Furthermore, sphingolipids have been implicated in Parkinson's disease (PD) and Huntington's disease (HD) [13]. For example, glucocerebrosidase mutations have been suggested to be risk factors for PD and other Lewy body disorders [14]. Fibroblasts of HD patients and the brain of HD animal models exhibit reduced GM1 ganglioside levels [15]. Lastly, changes in sphingolipids have also been associated with metabolic diseases that are caused by mutations of proteins involved in sphingolipid degradation. Storage of sphingolipids in these diseases results in global impairment of lysosomal function [16]. This in turn blocks lysosomal degradation of defective proteins and organelles.

While we are beginning to understand the molecular pathology of sphingolipid-related and other lysosomal storage diseases, the roles of sphingolipid metabolism in the progression of aging and neurodegeneration are still not clear. It was reported that disruption of the *Lass2* gene that encodes ceramide synthase 2 (CerS2), caused myelin degeneration, consistent with the restricted expression of this gene within the brain to oligodendrocytes, and secondary loss of cerebellar granule cells [17]. This finding demonstrates that reduction of sphingolipid levels is indeed pathogenic in the brain, at least in oligodendrocytes. Here we report that deficiency of *Lass 1* that encodes CerS1, a ceramide synthase that is predominantly expressed in neurons [18], [19], causes progressive Purkinje cell loss in mice. We also show that CerS1 plays a key role in ceramide biosynthesis in the brain, and loss of this protein dramatically impacts many aspects of sphingolipid homeostasis. Lastly, we find that loss of CerS1 leads to accumulation of lipofuscin that are associated with ubiquitylated proteins in many regions of the brain, suggesting that ceramide biosynthesis is critical for protein and organelle homeostasis. These data demonstrate that lipid biosynthesis defects in neurons can directly cause cell death. Furthermore, our results establish a causal link between lipid biosynthesis deficiencies and lipofuscin accumulation, and may provide a common mechanism for deposition of lipofuscin in aging and neurodegenerative diseases.

## Results

### Cerebellar Purkinje cell defects in the flincher mutant brain

The flincher (*fln*) mutation arose spontaneously in a colony of NOD.CB17-*Prkdc^scid^*/SzJ mice at The Jackson Laboratory. Mice homozygous for the *fln* mutation were hyperactive and generally smaller in body size beginning at postnatal day seven. By three weeks of age, the size difference between mutant mice and their littermates was very obvious. Although mutant mice continued to gain weight until reaching maturity, adult mutant mice progressively lost weight, like other ataxic mice. However, the lifespan of mutant mice was comparable to that of their wild type or heterozygous littermates despite their weight loss and severe ataxia.

**Figure 1:**
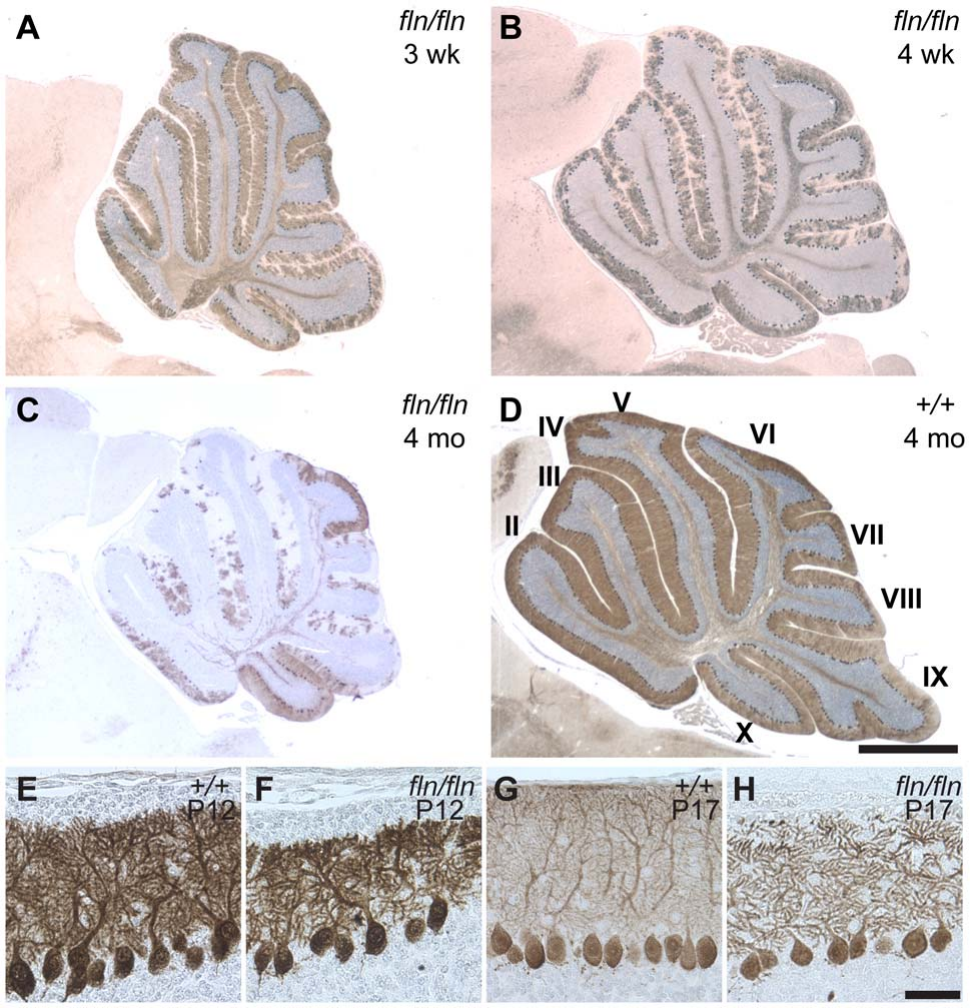
Progressive cerebellar Purkinje cell degeneration and abnormalities of Purkinje dendritic arbors in *fln/fln* mice. (A–D) Calbindin D-28 immunostained sagittal sections of cerebella from three-week-old (B), four-week-old (B) and four-month-old *fln/fln* (C), and four-month-old wild type (*+/+*, D) mice. Sections were counterstained with hematoxylin. Cerebellar lobules are indicated by Roman numerals. (E–H) Purkinje cells immunostained with calbindin D-28 from wild type (E, G) and *fln/fln* (F, H) mice at P12 (E, F) and P17 (G, H). Scale bars: 1 mm (A–D); 50 µm (E–H).

Flincher mutant mice displayed cerebellar ataxia beginning at three weeks, which was concomitant with degeneration of cerebellar Purkinje cells (Video S1, Figure 1A and data not shown). Neuron loss was progressive, and many Purkinje cells had degenerated in mutant mice by four months of age (Figure 1B–1D). Although the soma of Purkinje cells appeared normal in the mutant mice prior to three weeks of age, at postnatal day twelve (P12), Purkinje cell dendritic arbors were shorter than those in the wild type cerebellum (Figure 1E–1F). This reduction of dendritic arbor size was more pronounced by P17 (Figure 1G–1H). In addition, the reduced density of calbindin immunostaining in the molecular layer at P17 suggested that the complexity of higher order branches of mutant Purkinje cell dendritic arbors may also be reduced compared to that of wild type dendrites.

### The flincher mutation disrupts the *Lass1* gene

**Figure 2:**
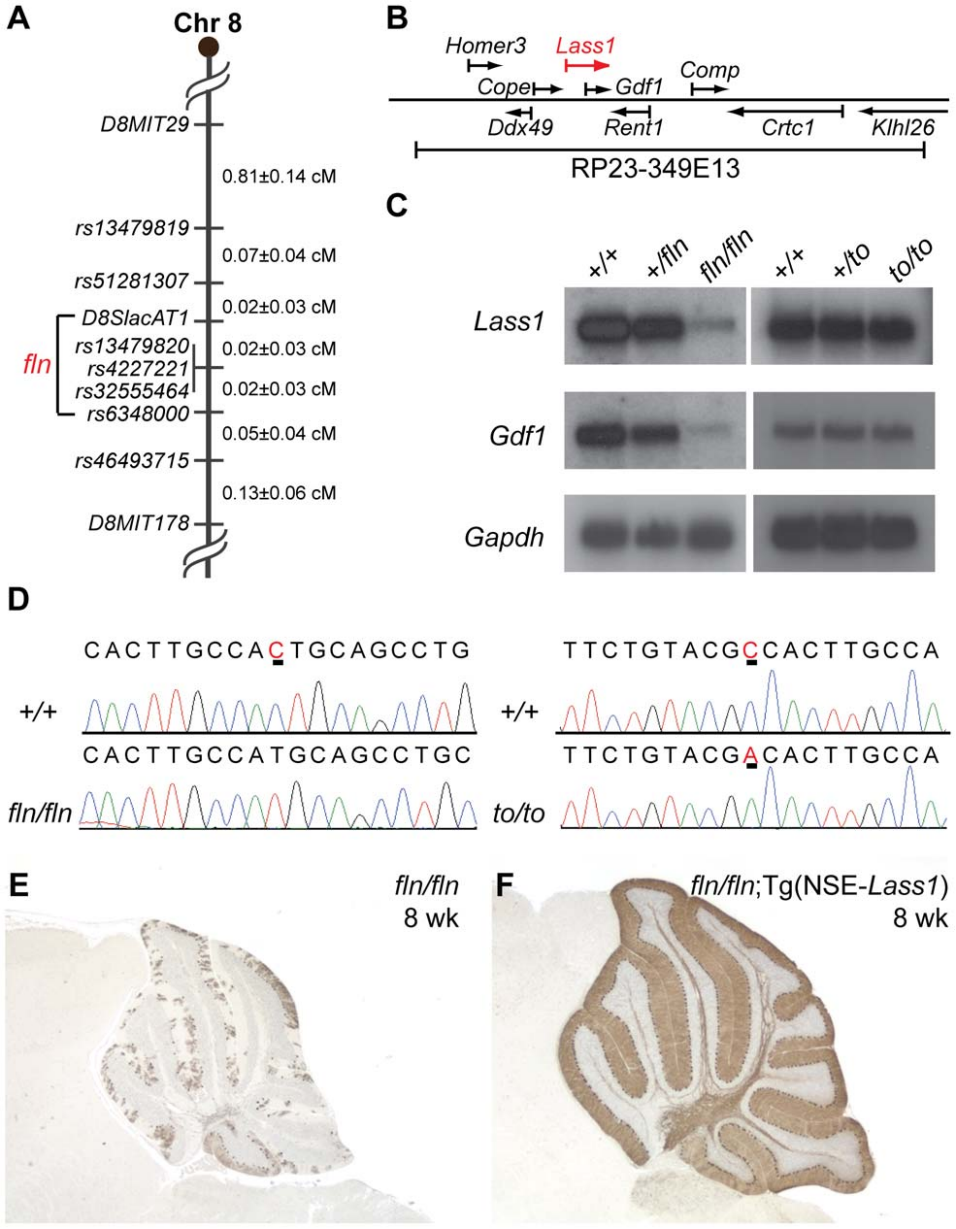
Genome mapping and identification of the flincher and toppler mutations. (A) The critical mapping interval of the *fln* mutation is between *D8SlacAT1* and *rs6348000*. (B) The genes encoded in BAC RP23-349E13 that was used to construct the *fln*-rescuing BAC transgenic line. (C) Northern blot analyses of *fln/fln* and *to/to* brain mRNA hybridized with probes to *Lass1* and *Gdf1*. Blots were re-hybridized with a *Gapdh* probe as a loading control. (D) Sequence chromatograms of wild type (*+/+*), *fln/fln* and *to/to Lass1* cDNA reveal a single nucleotide deletion (underlined in the wild type chromatogram) in *fln/fln* mice and a missense mutation (underlined) in *to/to* mice. (E–F) Calbindin D-28 immunohistochemistry of cerebella from 8-week-old *fln/fln* (E) and *fln/fln*; Tg(NSE-*Lass1*) (F) mice.

To identify the molecular defect in *fln* mutant mice, we crossed *fln/fln* and BALB/cByJ mice, and performed genome scans on F2 mice using sequence tagged site (STS) markers. This analysis localized the *fln* mutation to Chromosome 8. Fine mapping using these F2 mice and additional F2 mice from a *fln*×CAST/EiJ cross narrowed the *fln* mutation to a 0.04 cM (0.8 Mb) region between two single nucleotide polymorphisms (SNPs), *D8SlacAT1* and *rs6348000* (two recombinants/1984 F2 mice), containing 34 known protein-encoding genes (Figure 2A, Figure S1 and Figure S2A). To further define the mutant locus, transgenic mice carrying bacteria artificial chromosomes (BACs) containing genes from the *fln* critical interval were generated and crossed with mutant mice (Figure S2A). Among the three BACs used to make transgenic mice, only BAC RP23-349E13 was able to complement the *fln* mutant phenotype (Figure S2B–S2C). BAC RP23-349E13 contains nine known genes (Figure 2B) and the expression of one of these genes, *Lass1*, was greatly reduced in *fln/fln* brains (Figure 2C, left panels). Sequencing of *Lass1* RT-PCR products revealed a single nucleotide deletion in exon 5, which results in a frameshift mutation that likely leads to nonsense-mediated decay of the *Lass1* transcript in *fln* mutant brains (Figure 2D, left panels).

*Lass1* is transcribed as a part of an unusual bicistronic transcript that also encodes growth differentiation factor-1 (GDF1), a member of the TGF-ß family [20]. Thus, as expected, *Gdf1* expression is also reduced in *fln* mutant brains (Figure 2C, left panels). To determine if the *fln* phenotype is a result of loss of *Lass1* or of decreased *Gdf1* function, we performed cDNA transgenic complementation experiments. Transgenic mice expressing the *Lass1* cDNA under the control of the neuron-specific enolase (NSE) promoter were generated and mated with *fln* mutant mice to generate *fln/fln* mice carrying the *Lass1* transgene. These mice did not develop ataxia nor did they have Purkinje cell degeneration, demonstrating that expression of *Lass1* in neurons is sufficient to rescue *fln*-mediated Purkinje cell degeneration (Figure 2E–2F and data not shown). Therefore, loss of *Lass1*, not *Gdf1*, function underlies the neuropathology observed in *fln* mutant mice.

### Toppler (*to*) is a point mutation in the *Lass1* gene

Toppler (*to*), a spontaneous recessive mutation causing Purkinje cell degeneration beginning around three weeks after birth, was also mapped to the middle of Chromosome 8 [21]. Postnatal Purkinje cells in *to* mutant cerebella also display higher order dendritic branching pattern abnormalities. Based on the striking phenotypic similarity, we proposed that *to* and *fln* mutations were allelic and performed complementation tests. *Fln/+; to/+* compound heterozygous mice exhibited progressive ataxia beginning at three weeks of age (data not shown). These mice also had Purkinje cell degeneration that was indistinguishable from that was observed in mice homozygous for either the *to* or the *fln* mutation (data not shown), indicating that the *to* mutation also likely disrupts the *Lass1* gene. This inference was confirmed by the rescue of the *to* mutant phenotype by the *Lass1* cDNA transgene (Figure S3A–S3B). Although the RNA expression level of the bicistronic *Lass1-Gdf1* transcript was not affected by the *to* mutation (Figure 2C, right panels), sequencing of *Lass1* RT-PCR products from toppler mutants revealed a missense point mutation in exon 5 resulting in the change of residue Ala266 to Asp (Figure 2D, right panel, and Figure S4B). These results confirmed that the *Lass1* gene underlies the pathological changes in both *fln* and *to* mutant mice.

### The *fln* and *to* mutations disrupt ceramide synthase 1 enzymatic activity

**Figure 3:**
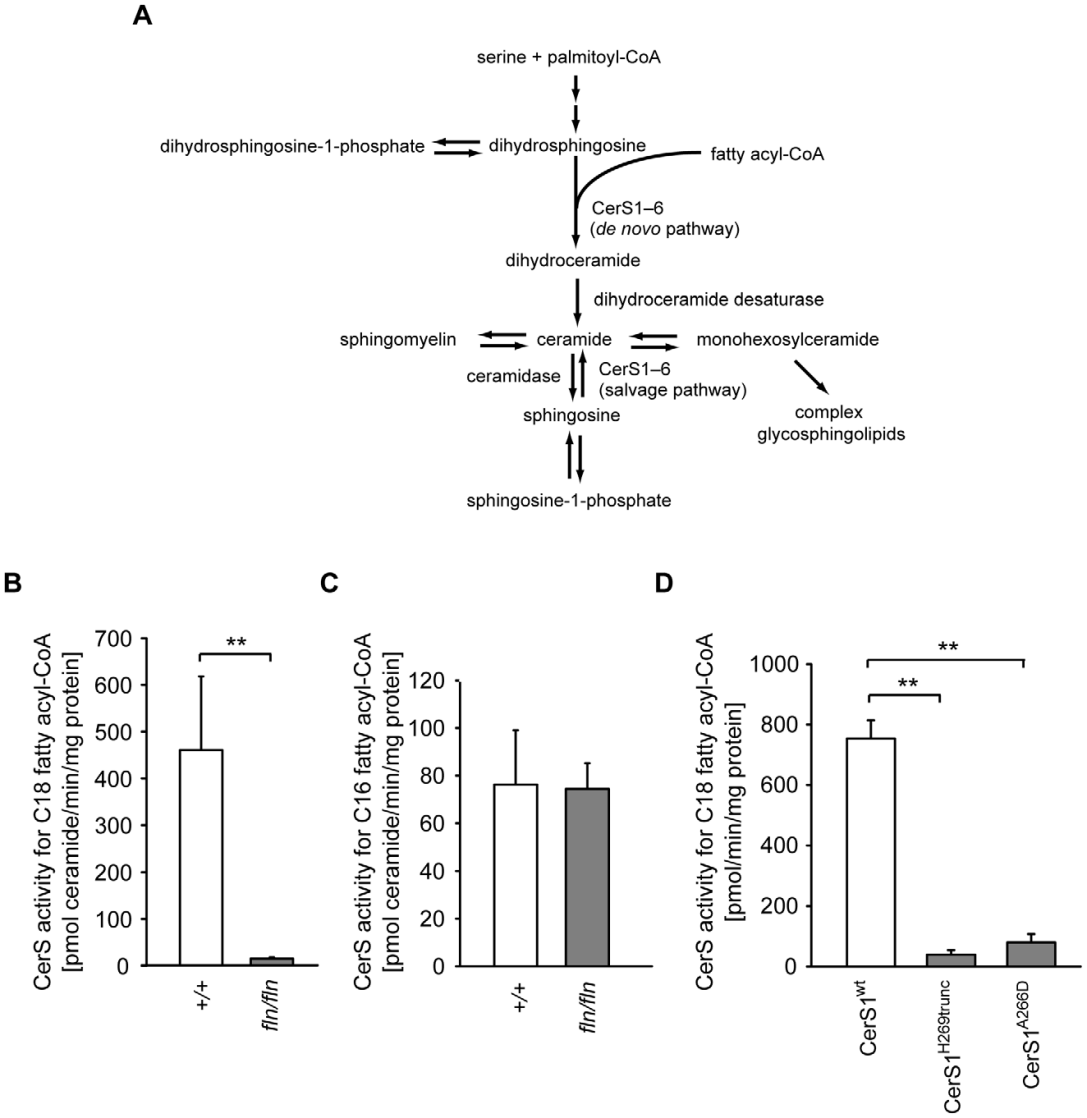
The *fln* and *to* mutations impair ceramide synthase activity of CerS1. (A) Schematic diagram of mammalian ceramide biosynthesis. (B–C) Ceramide synthase activities of brain microsomes from 12- to 13-day-old wild type (+/+, open bars; n = 5) and *fln/fln* mice (filled bars; n = 6). CerS activities for C_18_ fatty acyl-CoA (B) and C_16_ fatty acyl-CoA (C) are shown as mean ± SD. (D) *In vitro* C_18_ ceramide synthase activities of wild type and mutant CerS1 proteins. Full-length wild type CerS1 (CerS1^wt^), or CerS1^H269trunc^ (*Lass1^fln^*) or CerS1^A266D^ (*Lass1^to^*) were expressed in COS7 cells. Values are mean ± SD from three independent experiments. No significant difference of C_18_ ceramide synthase activity was observed between CerS1^H269trunc^ and CerS1^A266D^. **: *p*≤0.01.

The *Lass1* gene encodes the (dihydro)ceramide synthase CerS1, one of the six (dihydro)ceramide synthases (CerS1–CerS6) in mammals, encoded by *Lass1* to *Lass6*, respectively [22]. In the *de novo* pathway of ceramide biosynthesis, these enzymes catalyze the condensation of fatty acyl-CoA and dihydrosphingosine (dhS, also known as sphinganine) to produce dihydroceramide (Figure 3A). Dihydroceramide, in turn, is desaturated on the dhS backbone by dihydroceramide desaturase to form ceramide, which is the basic building block for complex sphingolipids. *In vitro* overexpression studies demonstrated that each of the six mammalian ceramide synthases has different fatty acyl-CoA substrate specificity and saturated C_18∶0_ fatty acyl-CoA is the preferred substrate for CerS1 [23]–[25]. These studies also demonstrated that CerS1 could also use C_16∶0_ and C_20∶0_ fatty acid-CoA, albeit inefficiently, but not the unsaturated C_18∶1_ fatty acyl-CoA that contains a Δ9 *cis* double bond [25].

Similar *in vitro* assays suggested that C18∶0 fatty acyl-CoA can also serve as a substrate for CerS5, a CerS with broader substrate specificity [25]. However, CerS5 is expressed at a lower level than that of CerS1 in the mouse brain [25]. Together these data suggested that the reduction of CerS1 function in the mouse brain would significantly decrease the activities of ceramide synthases that utilize C_18_ fatty acyl-CoA. To test this hypothesis, we analyzed ceramide synthase activity with three fatty acyl-CoA substrates, including C_18∶0_ fatty acyl-CoA, in microsomes prepared from wild type and mutant brain homogenates. The activity for C_18_ fatty acyl-CoA was reduced drastically in *fln*/*fln* brain microsomes, indicating that CerS1 is the major CerS in the brain using C_18_ fatty acyl-CoA (Figure 3B). No difference in ceramide synthase activity for C_16_ or C_24_ fatty acyl-CoA was observed between wild type and *fln* mutant brain microsomes, confirming the high specificity of CerS1 for C_18_ fatty acyl-CoA, and suggesting that the activities of other ceramide synthase isoenzymes present in the brain were not affected by the severely reduced CerS1 activity (Figure 3C and data not shown).

CerS1 is a multiple transmembrane domain protein and shares a conserved TRAM-LAG1-CLN8 (TLC) domain with other CerSs. The *fln* and *to* mutations reside between the last two predicted transmembrane domains. This region is within the TLC domain but outside of the LAG1 motif that was previously shown to be indispensible for the catalytic activity (Figure S4A) [26]. Mice homozygous for either mutation have very similar phenotypes, suggesting that the Ala266Asp *to* mutation may also result in a dramatic loss of CerS1 function. To test this possibility, plasmids encoding wild type and mutant CerS1 proteins, each with an N- terminal FLAG epitope, were transfected into COS7 cells. Microsomes prepared from transfected cells were assayed for CerS activity using C_18_ fatty acyl-CoA as a substrate, and the crude enzymatic activity was normalized to the levels of FLAG-tagged CerS1. Both *fln* and *to* mutations impart almost complete loss of CerS1 catalytic activity, demonstrating that the carboxyl end of the TLC domain, and residue Ala266 in particular, are indispensible for CerS1 function (Figure 3D).

**Figure 4:**
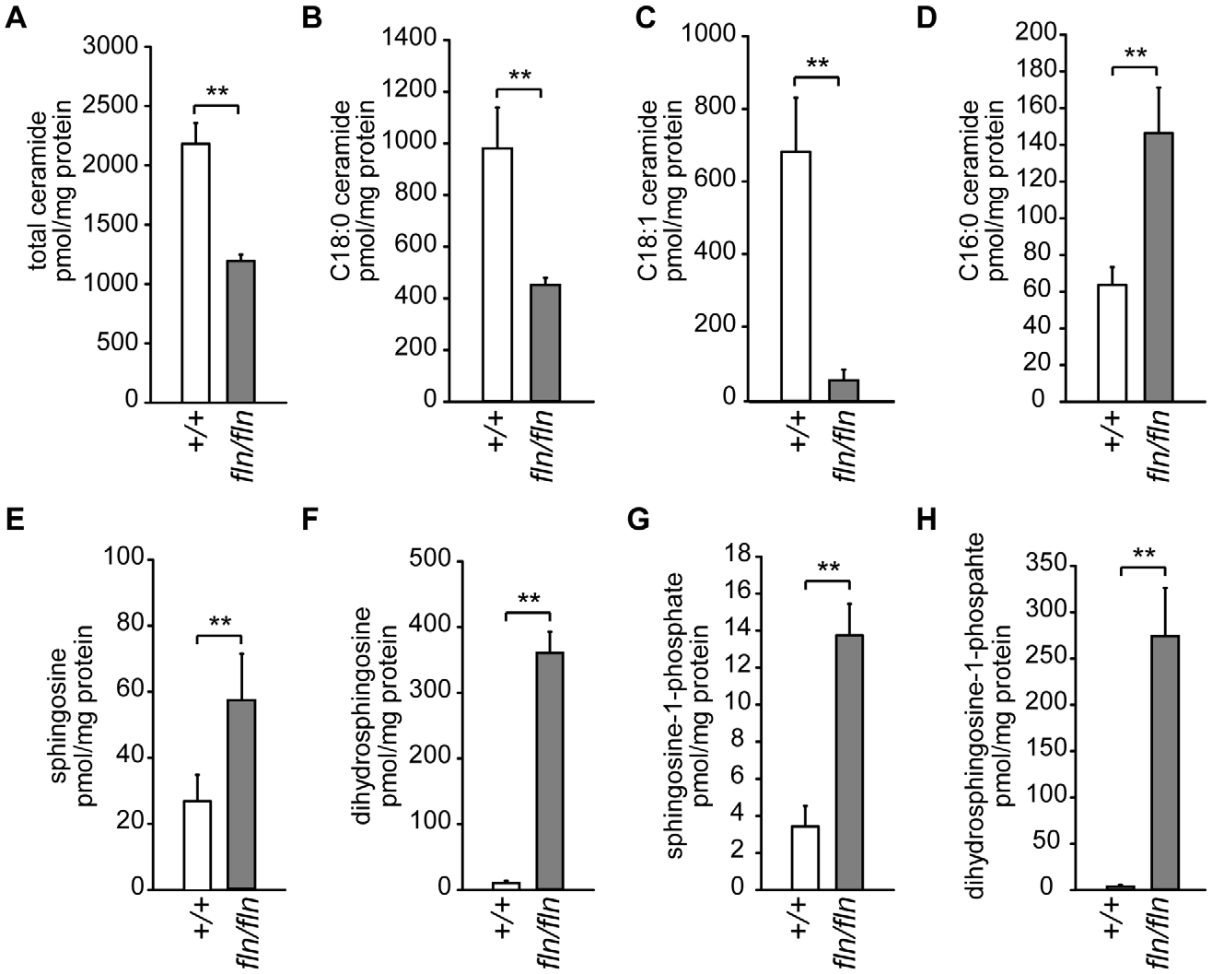
Alterations of sphingolipid homeostasis in *fln* mutant brains. Brain sphingolipid levels for selected sphingolipid species in 12- to 13-day-old wild type (+/+, open bars; n = 6) and *fln/fln* mice (filled bars; n = 5) were measured by mass spectrometry and normalized to protein concentration. Total ceramide (A), and C_18∶0_ (B), C_18∶1_ (C), and C_16∶0_ (D) ceramide species are shown. Sphingosine (E), dihydrosphingosine (F), sphingosine-1-phosphate (G), dihydrosphingosine-1-phosphate (H) levels are also shown. All values are mean ± SD. **: *p*≤0.01.

To investigate the effect of CerS1-deficiency on sphingolipid homeostasis in the brain, total lipids were extracted from wild type and *fln* mouse brains, and subjected to liquid chromatography coupled mass spectrometry (LC-MS). The total amount of ceramide was decreased by approximately 50%, confirming that CerS1 is a major CerS in the brain (Figure 4A). In line with the previous report on the preference of CerS1 for C_18∶0_ fatty acyl-CoA, C_18∶0_ ceramide was reduced approximately two fold in the mutant samples (Figure 4B). Although previous *in vitro* data demonstrated that unsaturated C_18∶1_ fatty acyl-CoA was not utilized by CerS1 [25], the level of C_18∶1_ ceramide was decreased more than five fold in the *fln* mutant brain suggesting that either CerS1 can utilize C_18∶1_ fatty acyl-CoA *in vivo*, or the C_18∶1_ side chain is formed by fatty acyl chain desaturation after ceramide biosynthesis (Figure 4C). Our results also confirm that C_18_ ceramide species are the dominant ceramide species in the mouse brain. Ceramide is the basic building block of complex sphingolipids, such as sphingomyelin and glycosphingolipids. In agreement with the reduced levels of C_18_ ceramide species, the amount of complex sphingolipids with C_18_ fatty-acyl groups was also reduced in the *fln/fln* brain (Figure S5A and data not shown).

Contrary to the reduction of C_18_ sphingolipids observed in the *fln* mutant brain, the amounts of other long-chain sphingolipids, i.e. sphingolipids with fatty acyl chain length moiety C_14_ and C_16_, were increased significantly, suggesting a compensatory mechanism (Figure 4D, Figure S5 and data not shown). This compensation was not likely due to an upregulation of CerS5 or CerS6 that utilize C_14_ and C_16_ fatty acyl-CoA for ceramide biosynthesis, given that we did not observe an increased ceramide synthase activity with C_16_ fatty acid CoA in *fln/fln* brain microsomes (Figure 3C). Rather, increased C_14_ and C_16_ sphingolipid levels were most likely the result of excessive substrate availability for CerS5 and CerS6. The excessive substrate availability for CerS5 may also underlie the similar levels of C_18_ dihydroceramide observed in wild type and *fln/fln* brains (Figure S5C).

Sphingolipids undergo constant synthesis and degradation. Ceramide can be generated by degradation of complex sphingolipids, and be degraded further by ceramidases to release the sphingoid base, sphingosine. Sphingosine, in turn, can be recycled and used as the sphingoid substrate in the salvage pathway of ceramide biosynthesis, which is also catalyzed by CerSs (Figure 3A). Thus, loss of CerS1 function could result in accumulation of sphingosine, and dhS, the sphingoid substrate of *de novo* ceramide biosynthesis. Indeed, LC-MS analyses of *fln/fln* brains revealed a 2–4 fold increase in sphingosine and a greater than 10 fold increase in dhS (Figure 4E–4F). The phosphorylated metabolites of sphingosine and dhS, sphingosine-1-phosphate (S1P) and dhS-1-phosphate (dhS1P) were also significantly increased in mutant brains (Figure 4G–4H). However, the accumulation of dhS and dhS1P was much more pronounced than that of sphingosine and S1P, suggesting that the *de novo* ceramide biosynthesis pathway is the dominant pathway for ceramide biosynthesis in the brain.

### Lipofuscin accumulates in CerS1 mutant brains

In normal aging brains and under some pathological conditions, undigested storage materials may accumulate in neurons to form autofluorecent deposits, termed lipofuscin. Based on the ultrastructure of lipofuscin, it has been suggested that lipofuscin production is a result of impaired autophagy of organelles, especially mitochondria [27]. Consistent with this hypothesis, mutations of several lysosomal proteins have been linked to the neuronal ceroid lipofuscinoses (CLNs), a group of infantile- to juvenile-onset neurodegenerative diseases that are characterized by abnormal lipofuscin accumulation [28]. However, the recent identification of two CLN proteins, CLN6 and CLN8, as polytopic membrane proteins on the endoplasmic reticulum suggests that non-lysosomal mechanisms may underlie lipofuscin accumulation in these diseases [29]–[31]. Although CLN8 does not show detectable ceramide synthase activity, ceramide profile changes have been observed in the brains of CLN8 patients [12]. In addition, overexpression of CLN8 or CerSs suppressed the growth phenotype of fibroblasts from patients with CLN9, the causal gene of which has yet to be identified [32]. These results indicate that defects in ceramide homeostasis may contribute to accumulation of lipofuscin.

**Figure 5:**
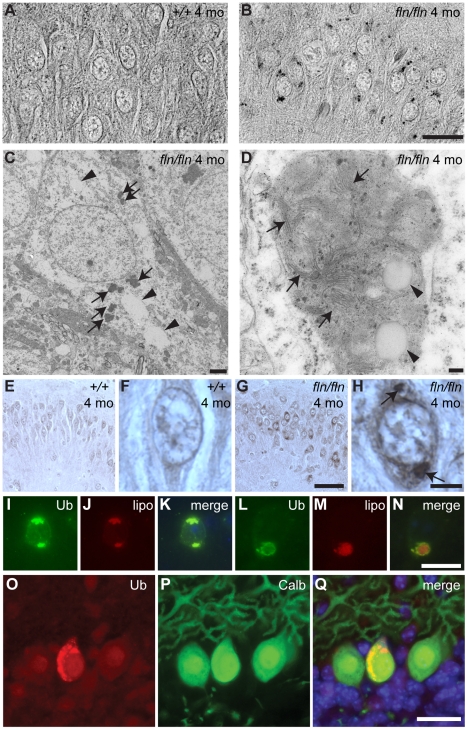
Lipofuscin and ubquitylated protein accumulation in CerS1-deficient neurons. (A–B) Sections of cerebella from four-month-old wild type (+/+, A) and *fln/fln* (B) mice were stained with Luxol Fast Blue. Images are converted to gray-scale for clear visualization of punctate staining. Images of the hippocampal CA3 region are shown. (C–D) Electron micrographs showing electron-dense deposits in *fln/fln* neurons at five months of age. An overview of a hippocampal neuron (C) showing vacuoles (arrowheads) and electron-dense deposits (arrows), and a lipofuscin structure from a cortical neuron (D) showing internal membrane structures (arrows) and vacuoles (arrowheads). (E–H) Ubiquitin immunohistochemistry of the hippocampal CA3 region from 4-month-old wild type (+/+, E, F) and *fln/fln* (G, H) mice. Details are shown in higher magnification (F, H). Conspicuous ubiquitylated accumulations in an *fln/fln* neuron are marked with arrows (H). (I–N) Ubiquitylated proteins (Ub; I, L) and autofluorescent lipofuscin (lipo; J, M) in a hippocampal neuron (I–K) and a cortical neuron (L–N) of a four-month-old *fln/fln* mouse. Merged images (K, N) show the relative locations of the ubiquitin immunofluorescent stain and autofluorescence. (O–Q) Ubiquitin-positive inclusions in Purkinje cells of three-week old *fln/fln* mice. Sections were incubated with an antibody against ubiquitin (O) and calbindin D-28 (P). Merged images are shown (Q), with Hoescht dye counterstaining. Scale bars: 20 µm (A–B); 2 µm (C); 100 nm (D); 50 µm (E, G); 20 µm (F, H, I–N); 25 µm (O–Q).

To directly test whether a deficiency of ceramide biosynthesis can induce lipofuscin accumulation, we examined adult *fln* and *to* mutant brains. Although autofluorescent deposits were observed sporadically in neurons of four-month-old wild type mice, deposits were widespread in many brain regions in mutant mice, with the highest abundance in deep cerebellar nuclei, pons, medulla, anterior olfactory nuclei, layer IV to layer VI of the cerebral cortex, and the CA3 region of the hippocampus (data not shown). A similar pattern of deposits was observed in sections of mutant brains that were stained with Luxol fast blue, a compound that stains myelin and lipofuscin (Figure 5A–5B). To ascertain that these observed deposits are indeed lipofuscin, we examined their fine structure. Electron microscopy revealed the presence of many vacuoles and electron dense structures in the cytoplasm of mutant neurons (Figure 5C). Higher magnification revealed membranes and vacuoles within these dense structures, as typically seen in neuronal lipofuscin (Figure 5D).

Lipofuscin often contains both lipids and ubiquitylated proteins [1]. To test whether ubiquitylated proteins also accumulate in the lipofuscin in CerS1 mutant brains, we performed immunohistochemistry using an antibody against ubiquitin. Indeed, ubiquitin-positive deposits were observed in the same types of neurons harboring lipofuscin, including cerebral cortical neurons, hippocampal CA3 neurons, and neurons in deep cerebellar nuclei, pons, medulla and anterior olfactory nuclei (Figure 5E–5H, and data not shown). Although the autofluorescent deposits are visible across a wide spectrum of light, the intensity of their fluorescence is lower than that of the fluorescent molecule-conjugated secondary antibodies used for ubiquitin immunofluorescence, allowing simultaneous assessment of lipofuscin and ubiquitin-positive puncta. We found that ubiquitylated proteins and autofluorescent deposits were largely colocalized or closely associated (Figure 5I–5N). While lipofuscin accumulated in many neurons in the mutant brain, we did not observe overt death of neurons other than Purkinje cells, although we cannot rule out more subtle changes in the survival of mutant neurons. However, as evidenced by the hyperactivity of *CerS1* mutant mice, it is likely that the function of additional neurons is impaired in these mice.

Although lipofuscin deposition was much more pronounced in brains of older mutant mice, we also observed weak autofluorescent deposits containing ubiquitin-positive puncta in the soma and swollen dendrites of a small number of Purkinje cells in 3-week old mutant, but not wild type, mice (Figure 5O–5Q and data not shown). This result suggests that CerS1-deficiency also causes accumulation of lipofuscin-like structures in at least some Purkinje cells.

## Discussion

Alterations of sphingolipids have been observed in many neurodegenerative disorders. However, the contribution of these changes to disease pathogenesis is not clear [5]. We have uncovered two spontaneous mutants with progressive degeneration of cerebellar Purkinje neurons and widespread lipofuscin accumulation. By positional cloning, we identified the mutations as alleles of the *Lass1* gene, which encodes ceramide synthase 1 (CerS1), one of the six ceramide synthases in mammals. Our data demonstrate that deficiency of CerS1/LASS1 leads to dramatic alterations in the levels of sphingolipids in the brain, degeneration of cerebellar Purkinje neurons, and widespread lipofuscin accumulation.

CerS1 is specifically expressed in neurons in the brain and has been shown to produce C_18∶0_ ceramide species *in vitro* or in cultured cells [18], [19], [23]–[25]. In the brain of *Lass1* mutant mice, C_18_ sphingolipid species were significantly reduced, confirming the role of CerS1 in C_18_ ceramide biosynthesis *in vivo*. Loss of CerS1 also led to a decrease in total ceramide, in agreement with C_18_ ceramide as a major ceramide in the adult brain [17]. However these sphingolipid decreases were accompanied by an increase in the steady state levels of C_14_ and C_16_ sphingolipid species. In addition, sphingoid bases and their phosphorylated metabolites were drastically increased in the mutant brain.

In contrast to previous reports suggesting that ceramide is proapoptotic *in vitro* and can mediate both stress-induced intrinsic and death receptor-mediated extrinsic apoptosis [8], [33], our data suggest that reduction of ceramide or complex sphingolipids results in progressive neuron death, particularly Purkinje cell loss. Intracerebroventricular administration of global ceramide inhibitors has been reported to cause acute neurodegeneration [34]. Contrary to *in vitro* data, this finding, together with our results, demonstrate that decreases in ceramide synthesis can induce neuron death *in vivo*. Sphingolipid homeostasis is critically balanced in the cell under normal conditions, and thus either increases or decreases of ceramide could be detrimental to the cell. Alternatively, ceramide species with different fatty acyl chains may play distinct physiological roles. For example, loss of one of the two worm ceramide synthases, which produce different ceramide species, resulted in opposite outcomes in *C. elegans* under hypoxic conditions [35]. Perhaps C_18_ ceramide, or some of its derived sphingolipids such as C_18_-sphingomyelin or C_18_-cerebrosides, act in a prosurvival fashion in neurons, whereas an increase in other ceramide species, such as C_16_ ceramide, may be apoptotic [35]. Lastly, Purkinje neuron loss may be due to an increase in the sphingoid bases that normally serve as substrates for CerS1. Sphingoid bases, particularly their phosphorylated metabolites, are potent signaling molecules and have been proposed to regulate many signal transduction pathways [7]. Thus, elevation of these molecules, particularly dhS and dhS1P, which are increased over ten fold in the CerS1-deficient brain, may impair normal functions of sphingolipid signaling, leading to Purkinje cell death.

Consistent with previous reports demonstrating that sphingolipid biosynthesis is crucial for dendritic development in cultured Purkinje or hippocampal neurons [36], [37], we observed shortened dendritic arbors and decreased density of calbindin immunostaining in the molecular layer in CerS1-deficient cerebella suggesting that dendritic development of mutant Purkinje cells was abnormal. The role of sphingolipids in dendritic development is not clear, but may involve sphingolipid-rich lipid rafts, which are implicated in activity-dependent dendritic development and maintenance of dendritic spines in *in vitro* experiments [38], [39]. Dendritic dysfunction or morphological anomalies are often associated with neurodegeneration [40]. While we cannot rule out a secondary cause for abnormalities in Purkinje cell dendrites in CerS1 mutant mice, it is possible that defects in dendritic development also contribute to death of these neurons.

In addition to Purkinje cell death, our data clearly demonstrate that deficiency of ceramide biosynthesis can cause the accumulation of lipofuscin, which is often observed in aging brains and in some neurodegenerative diseases. Lipofuscin is known to contain both lipids and proteins that may originate from membrane bound organelles such as mitochondria [1]. Accumulation of lipofuscin has been linked to reduced lysosomal hydrolytic capacity, based on observations of lipofuscin deposition in mutants with deficiencies in lysosomal proteins [1], [28]. Both increases and decreases in ceramide have been shown to induce autophagy, suggesting that the balance of sphingolipid homeostasis is important for autophagy and/or lysosomal functions [41], [42]. Alternatively, conditions that increase the load on lysosomes beyond their capacity may also underlie lipofuscin formation. Given the importance of sphingolipids in protein targeting and as components of membranes, reduced sphingolipid levels may increase the demand on, and/or decrease the capacity of lysosomal function by affecting both lysosomal and non-lysosomal membrane dynamics, and targeting of membrane proteins. Increased load of defective membrane organelles and mislocalized membrane proteins could exceed lysosomal hydrolytic capacity of neurons, resulting in lipofuscin formation.

Previous reports suggest ceramide might also be important during aging [43]. One of the yeast genes encoding ceramide synthases, *LAG1* (**l**ongevity **a**ssurance gene 1), was identified in a screen for genes whose expression changed over yeast replicative cycles [44]. Deletion of *LAG1* was associated with an increase of replicative life span in yeast. However, a *LASS1* variant exhibiting increased expression, when combined with specific *HRAS1* and *APOE* haplotypes, was suggested to contribute to healthy aging and survival in a human population [45]. This finding suggests that appropriately increased ceramide levels might be beneficial to some cells in aging animals. Inversely, given the specificity of Purkinje cell loss observed in CerS1-deficient mice, decreased ceramide levels appear more detrimental to neurons, and accelerate aging phenotypes, including lipofuscin accumulation. Thus our results demonstrate that in addition to neurodegeneration, alteration of ceramide levels can accelerate some aspects of aging. More research is needed to elucidate the role of ceramide and other sphingolipids in aging and neurodegeneration.

## Materials and Methods

### Ethics statement

All experiments with mice have been approved by The Jackson Laboratory Animal Care and Use Committee according to relevant national and international guidelines.

### DNA oligos and genotyping assays

Oligos used for plasmid construction and genotyping are listed in Table S1. Genotyping assays are described in detail in Text S1.

### Mouse strains and plasmids

The *fln* and *to* mutations were maintained on a NOD/SzJ background and a FVB/N background, respectively. Bacterial artificial chromosome (BAC) transgenic mouse lines were generated by injection of purified BAC DNA into C57BL/6J pronuclei and maintained on the same background. To generate *Lass1* cDNA transgenic lines, *Lass1* cDNA was amplified from a mouse cDNA library plasmid pME18-FL3-LASS1 (Open Biosystems) with primers LZO455 and LZO456, and inserted into pNSE-Ex4N1 at the *Bam*HI and *Spe*I sites, downstream of the neuron-specific enolase 2 gene (*Nse2*) promoter. The plasmid pNSE-Ex4N1 is a modified version of pNSE-Ex4, which was described previously [46], with *Bam*HI, *Not*I and *Spe*I sites on an adapter inserted into the *Hind*III site of pNSE-Ex4. A *Sal*I fragment containing the *Lass1* cDNA, the *Nse2* promoter and an SV40 polyadenylation sequence was used for pronuclear microinjection. For expression of full-length wild type LASS1 protein in cultured mammalian cells, the *Lass1* coding region was recovered from the pNSE-Ex4N1-LASS1 plasmid by restriction digestion with *Bam*HI and *Eco*RI, and was inserted into pCMV-3Tag-1A (Stratagene), in frame with a 3xFLAG epitope. For expression of mutant LASS1 proteins, *Lass1* fragments were amplified from poly A^+^ RNA isolated from *fln/fln* or *to/to* mutant brains, using primers LZO605 and LZO606. The FLAG epitope-tagged wild type LASS1 construct was cleaved with *Bsr*GI to swap an internal fragment with the fragments containing the mutations.

### Genome mapping

Homozygous *fln* mice were crossed with BALB/cByJ mice, and F1 heterozygotes were intercrossed to generate F2 mice. Genome scans using polymorphic microsatellite markers were performed on DNA from ten affected and ten unaffected F2 mice. To fine map the mutation, 951 F2 mice were analyzed using polymorphic microsatellite markers and single nucleotide polymorphisms (SNPs). To further narrow the critical interval, *fln/fln* and CAST/EiJ were crossed, and F1 heterozygotes were intercrossed to generate F2 mice. 1033 F2 mice were analyzed.

### Preparation of RNA and northern blot analyses

Total brain RNA preparation, poly-A^+^ RNA selection and northern blot analyses were performed as described [47]. To generate northern blot probes, fragments of *Lass1* and *Gdf1* coding sequences were amplified with oligos LZO463 and LZO426, and oligos LZO445 and LZO446, respectively.

### Histology and immunohistochemistry

Immunohistochemical studies were performed as described [48]. Briefly, mice were intracardically perfused and brains were postfixed before dehydration and embedding in paraffin. After antigen retrieval in 0.01 M citrate buffer (pH 6), sections were incubated at 4°C overnight with mouse monoclonal antibody against calbindin D-28 (Sigma-Aldrich, 1∶1000) or rabbit polyclonal antibody against ubiquitin (DAKO, 1∶200) in phosphate-buffered saline with 0.03% Triton and 5% normal donkey serum. Colorimetric Calbindin D-28 immunohistochemistry was performed as described [49]. Fluorescent detection was performed with Alexa Fluor-conjugated secondary antibodies (Invitrogen, 1∶200). Autofluorescence was quenched by incubation with 0.01% Sudan Black in 70% ethanol, and some sections were counterstained with Hoescht 33258. To detect autofluorescent deposits, deparaffinated sections were mounted with Fluoromount-G (Southern Biotech) without incubation with Sudan Black.

For electron microscopy, mice were intracardically perfused with a mixture of 1.2% paraformaldehyde and 0.8% glutaraldehyde, and brains were postfixed and processed for transmission electron micrography using standard procedures [50].

### Cell culture and transfection

COS7 cells were cultured in Dulbecco's Modified Eagle's Medium (Invitrogen) supplemented with 10% Fetal Bovine Serum (Hyclone). Transfections were performed using Lipofectamine 2000 (Invitrogen) according to the manufacturer's protocol. Medium was replaced with fresh medium 12 hours after the transfection, and cells were harvested 48 hours after transfection.

### Microsomal preparation and *in vitro* ceramide synthase assays

For microsomal preparation from mouse brains, the tissue was homogenized in 20 mM HEPES buffer (pH 7.4, 2 mM KCL, 2 mM MgCl_2_, 250 mM sucrose) supplemented with protease inhibitors (Sigma) with a Tissue Tearor homogenizer (Biospec Products). For microsomal preparation from culture cells, harvested cells were lysed in 20 mM HEPES buffer (see above) with a 30-gauge syringe. Tissue homogenates or cell lysates were subjected to centrifugation at 1,000×g for 5 min at 4°C to remove unbroken tissue debris or intact cells and nuclei. The supernatants were subjected to centrifugation at 10,000×g for 10 min at 4°C to remove mitochondria. The supernatants were ultracentrifuged at 100,000×g for 1 h at 4°C to collect microsomes, which were re-suspended in HEPES buffer (see above), and protein concentrations were measured with the Bradford method (BioRad). For *in vitro* ceramide synthase assays, a 100 µL reaction mixture containing 15 µM C_17_ sphingosine (Avanti Polar Lipids) and 50 µM C_16_, C_18_ or C_24_ fatty acid CoA (Avanti Polar Lipids) in 25 mM potassium phosphate buffer (pH 7.4) was pre-warmed at 37°C for 5 min. The enzymatic reaction was initiated by adding microsomes (15 µg) to the reaction mixture, which was incubated at 37°C for 15 min, and the reaction was terminated by adding 2 mL extraction solvent (ethyl acetate/iso-propanol/water at 60/30/10 v/v/v), supplemented with d_13_/C_16_ ceramide and d_13_/C_22_ ceramide as internal standards for mass spectrometry analyses.

### Lipid extraction and liquid chromatography/mass spectrometry (LC/MS) analyses of sphingolipids

Lipid extractions and LC/MS analyses were performed as described previously [51]. Briefly, samples were fortified with internal standards. Lipids were extracted twice with 2 ml ethyl acetate/isopropanol/water (60/30/10 v/v) solvent system, dried under a stream of nitrogen, re-suspended into 150 µL 1 mM NH_4_COOH in 0.2% HCOOH in methanol, and analyzed by LC/MS. LC/MS analyses of sphingolipids were performed on a Thermo Finnigan TSQ 7000 triple quadrupole mass spectrometer, operating in a Multiple Reaction Monitoring positive ionization mode. Sphingolipid levels in the brain homogenates were normalized to protein concentration measured by the Bradford method.

### Western blot

Protein samples were separated on SDS gels (BioRad) and transferred to a nitrocellulose membrane (Amersham Biosciences) using standard techniques [52]. FLAG-tagged proteins were detected using M2 mouse monoclonal antibody against FLAG (Sigma). Mouse monoclonal N^+^/K^+^-ATPase antibody (Abcam) was used as a loading control. Primary antibodies were detected with an appropriate secondary antibodies conjugated with horseradish peroxidase, and detected with ECL (Amersham Pharmacia).

### Data analyses

*In vitro* ceramide synthase assays and mass spectrometry data were analyzed with Sigma Plot. Paired *t*-tests were performed.

## Supporting Information

Figure S1 Haplotypes of selected recombinants used to refine the critical interval of the *fln* mutation. F2 recombinants from NOD.CB17-*Prkdc^scid^/J-fln*×BALB/cJ and NOD.CB17-*Prkdc^scid^/J-fln*×CAST/EiJ crosses are shown with the number of recombinants with each haplotype indicated.
(TIF)

Figure S2 BAC rescue of the *fln* mutation. (A) The critical interval of the flincher mutation. The three BAC clones used in the BAC complementation experiments are shown. (B–C) Calbindin-D28 immunostaining of cerebellar sections from a seven-week-old *fln/fln* mouse and an age-matched *fln/fln* littermate carrying the BAC RP23-349E13 transgene. Scale bar: 1 mm (B–C).
(TIF)

Figure S3 A neuron-specific *Lass1* cDNA transgene suppresses the toppler mutation. Calbindin D-28 immunohistochemistry of cerebellar sections from eight-week-old *to/to* (A) and *to/to*; Tg(NSE-*Lass1*) (B) mice. Scale bar: 1 mm.
(TIF)

Figure S4 The *fln* and the *to* mutations are in the conserved TLC domain. (A) Predicted secondary structure of the mouse CerS1. Transmembrane domains were predicted with the TMPred server (http://www.ch.embnet.org/software/TMPRED_form.html) and combined with topological data of yeast ceramide synthases [53]. The *to* and the *fln* mutations are marked with arrowheads. The TLC domain is indicated in light green, and the LAG1 motif in the TLC domain is shaded in darker green. (B) Alignment of CerS1 orthologs using COBALT (http://www.ncbi.nlm.nih.gov/tools/cobalt/). Identical or similar residues across all species are colored red or orange, respectively. Residues conserved in vertebrates are indicated in yellow. Transmembrane domains were marked according to the mouse CerS1 sequence. Arrowheads denote the *fln* and the *to* mutations. Residues that are identical or conserved in mouse CerS1–CerS6 are marked with filled and open circles, respectively. Sequences used for analysis are NP_067090 (human), NP_619588 (mouse), NP_001037695 (rat), CAK11083 (Zebra fish), NP_727075 (*Drosophila*, fly), and NP493403 (*C. elegans*, worm).
(TIF)

Figure S5 Mass spectrometry data showing profound changes of sphingolipids in the *fln* mutant brain. Brain sphingolipid levels in 12- to 13-day-old wild type (+/+, open bars; n = 5) and *fln/fln* mouse brains (filled bars; n = 6) were measured by mass spectrometry and normalized to protein concentration. (A) Abundance of sphingomyelin with different fatty acyl chain moieties. (B) Abundance of ceramide with different fatty acyl chain moieties. Note that C_16_ and C_18_ ceramide species are shown in Figure 4, and are not included here. (C) Abundance of dihydroceramide with different fatty acyl chain moieties. All values are mean ± SD. *: *p*≤0.05; **: *p*≤0.01.
(TIF)

Table S1 Primers used to construct plasmids or used for genotyping.
(DOC)

Text S1 Genotyping assays.
(DOC)

Video S1 Mice homozygous for the *fln* mutation display severe cerebellar ataxia. A four-month old *fln/fln* and an age-matched wild type (+/+) littermate are shown.
(MP4)

## References

[1] Jung T, Bader N, Grune T (2007). Lipofuscin: formation, distribution, and metabolic consequences.. Ann N Y Acad Sci.

[2] Selkoe DJ (2003). Folding proteins in fatal ways.. Nature.

[3] Ross CA, Poirier MA (2005). Opinion: What is the role of protein aggregation in neurodegeneration?. Nat Rev Mol Cell Biol.

[4] Jung T, Catalgol B, Grune T (2009). The proteasomal system.. Mol Aspects Med.

[5] Ben-David O, Futerman AH (2010). The role of the ceramide acyl chain length in neurodegeneration: involvement of ceramide synthases.. Neuromolecular Med.

[6] Cutler RG, Kelly J, Storie K, Pedersen WA, Tammara A (2004). Involvement of oxidative stress-induced abnormalities in ceramide and cholesterol metabolism in brain aging and Alzheimer's disease.. Proc Natl Acad Sci U S A.

[7] Hannun YA, Obeid LM (2008). Principles of bioactive lipid signalling: lessons from sphingolipids.. Nat Rev Mol Cell Biol.

[8] Lahiri S, Futerman AH (2007). The metabolism and function of sphingolipids and glycosphingolipids.. Cell Mol Life Sci.

[9] Mielke MM, Lyketsos CG (2010). Alterations of the sphingolipid pathway in Alzheimer's disease: new biomarkers and treatment targets?. Neuromolecular Med.

[10] Wang G, Silva J, Dasgupta S, Bieberich E (2008). Long-chain ceramide is elevated in presenilin 1 (PS1M146V) mouse brain and induces apoptosis in PS1 astrocytes.. Glia.

[11] Haughey NJ, Cutler RG, Tamara A, McArthur JC, Vargas DL (2004). Perturbation of sphingolipid metabolism and ceramide production in HIV-dementia.. Ann Neurol.

[12] Hermansson M, Kakela R, Berghall M, Lehesjoki AE, Somerharju P (2005). Mass spectrometric analysis reveals changes in phospholipid, neutral sphingolipid and sulfatide molecular species in progressive epilepsy with mental retardation, EPMR, brain: a case study.. J Neurochem.

[13] Piccinini M, Scandroglio F, Prioni S, Buccinna B, Loberto N (2010). Deregulated sphingolipid metabolism and membrane organization in neurodegenerative disorders.. Mol Neurobiol.

[14] Lwin A, Orvisky E, Goker-Alpan O, LaMarca ME, Sidransky E (2004). Glucocerebrosidase mutations in subjects with parkinsonism.. Mol Genet Metab.

[15] Maglione V, Marchi P, Di Pardo A, Lingrell S, Horkey M (2010). Impaired ganglioside metabolism in Huntington's disease and neuroprotective role of GM1.. J Neurosci.

[16] Sabourdy F, Kedjouar B, Sorli SC, Colie S, Milhas D (2008). Functions of sphingolipid metabolism in mammals–lessons from genetic defects.. Biochim Biophys Acta.

[17] Imgrund S, Hartmann D, Farwanah H, Eckhardt M, Sandhoff R (2009). Adult ceramide synthase 2 (CERS2)-deficient mice exhibit myelin sheath defects, cerebellar degeneration, and hepatocarcinomas.. J Biol Chem.

[18] Laviad EL, Albee L, Pankova-Kholmyansky I, Epstein S, Park H (2008). Characterization of ceramide synthase 2: tissue distribution, substrate specificity, and inhibition by sphingosine 1-phosphate.. J Biol Chem.

[19] Becker I, Wang-Eckhardt L, Yaghootfam A, Gieselmann V, Eckhardt M (2008). Differential expression of (dihydro)ceramide synthases in mouse brain: oligodendrocyte-specific expression of CerS2/Lass2.. Histochem Cell Biol.

[20] Lee SJ (1991). Expression of growth/differentiation factor 1 in the nervous system: conservation of a bicistronic structure.. Proc Natl Acad Sci U S A.

[21] Duchala CS, Shick HE, Garcia J, Deweese DM, Sun X (2004). The toppler mouse: a novel mutant exhibiting loss of Purkinje cells.. J Comp Neurol.

[22] Pewzner-Jung Y, Ben-Dor S, Futerman AH (2006). When do Lasses (longevity assurance genes) become CerS (ceramide synthases)?: Insights into the regulation of ceramide synthesis.. J Biol Chem.

[23] Riebeling C, Allegood JC, Wang E, Merrill AH, Futerman AH (2003). Two mammalian longevity assurance gene (LAG1) family members, trh1 and trh4, regulate dihydroceramide synthesis using different fatty acyl-CoA donors.. J Biol Chem.

[24] Venkataraman K, Riebeling C, Bodennec J, Riezman H, Allegood JC (2002). Upstream of growth and differentiation factor 1 (uog1), a mammalian homolog of the yeast longevity assurance gene 1 (LAG1), regulates N-stearoyl-sphinganine (C18-(dihydro)ceramide) synthesis in a fumonisin B1-independent manner in mammalian cells.. J Biol Chem.

[25] Mizutani Y, Kihara A, Igarashi Y (2005). Mammalian Lass6 and its related family members regulate synthesis of specific ceramides.. Biochem J.

[26] Spassieva S, Seo JG, Jiang JC, Bielawski J, Alvarez-Vasquez F (2006). Necessary role for the Lag1p motif in (dihydro)ceramide synthase activity.. J Biol Chem.

[27] Sulzer D, Mosharov E, Talloczy Z, Zucca FA, Simon JD (2008). Neuronal pigmented autophagic vacuoles: lipofuscin, neuromelanin, and ceroid as macroautophagic responses during aging and disease.. J Neurochem.

[28] Jalanko A, Braulke T (2009). Neuronal ceroid lipofuscinoses.. Biochim Biophys Acta.

[29] Heine C, Koch B, Storch S, Kohlschutter A, Palmer DN (2004). Defective endoplasmic reticulum-resident membrane protein CLN6 affects lysosomal degradation of endocytosed arylsulfatase A.. J Biol Chem.

[30] Mole SE, Michaux G, Codlin S, Wheeler RB, Sharp JD (2004). CLN6, which is associated with a lysosomal storage disease, is an endoplasmic reticulum protein.. Exp Cell Res.

[31] Lonka L, Kyttala A, Ranta S, Jalanko A, Lehesjoki AE (2000). The neuronal ceroid lipofuscinosis CLN8 membrane protein is a resident of the endoplasmic reticulum.. Hum Mol Genet.

[32] Schulz A, Mousallem T, Venkataramani M, Persaud-Sawin DA, Zucker A (2006). The CLN9 protein, a regulator of dihydroceramide synthase.. J Biol Chem.

[33] Taha TA, Mullen TD, Obeid LM (2006). A house divided: ceramide, sphingosine, and sphingosine-1-phosphate in programmed cell death.. Biochim Biophys Acta.

[34] Osuchowski MF, Edwards GL, Sharma RP (2005). Fumonisin B1-induced neurodegeneration in mice after intracerebroventricular infusion is concurrent with disruption of sphingolipid metabolism and activation of proinflammatory signaling.. Neurotoxicology.

[35] Menuz V, Howell KS, Gentina S, Epstein S, Riezman I (2009). Protection of C. elegans from anoxia by HYL-2 ceramide synthase.. Science.

[36] Furuya S, Ono K, Hirabayashi Y (1995). Sphingolipid biosynthesis is necessary for dendrite growth and survival of cerebellar Purkinje cells in culture.. J Neurochem.

[37] Schwarz A, Futerman AH (1998). Inhibition of sphingolipid synthesis, but not degradation, alters the rate of dendrite growth in cultured hippocampal neurons.. Brain Res Dev Brain Res.

[38] Takemoto-Kimura S, Ageta-Ishihara N, Nonaka M, Adachi-Morishima A, Mano T (2007). Regulation of dendritogenesis via a lipid-raft-associated Ca2+/calmodulin-dependent protein kinase CLICK-III/CaMKIgamma.. Neuron.

[39] Hering H, Lin CC, Sheng M (2003). Lipid rafts in the maintenance of synapses, dendritic spines, and surface AMPA receptor stability.. J Neurosci.

[40] Lin RC, Matesic DF, Connor JA (1997). The role of dendritic dysfunction in neurodegeneration.. Ann N Y Acad Sci.

[41] Edinger AL (2009). Starvation in the midst of plenty: making sense of ceramide-induced autophagy by analysing nutrient transporter expression.. Biochem Soc Trans.

[42] Spassieva SD, Mullen TD, Townsend DM, Obeid LM (2009). Disruption of ceramide synthesis by CerS2 down-regulation leads to autophagy and the unfolded protein response.. Biochem J.

[43] Costantini C, Kolasani RM, Puglielli L (2005). Ceramide and cholesterol: possible connections between normal aging of the brain and Alzheimer's disease. Just hypotheses or molecular pathways to be identified?. Alzheimers Dement.

[44] D'Mello NP, Childress AM, Franklin DS, Kale SP, Pinswasdi C (1994). Cloning and characterization of LAG1, a longevity-assurance gene in yeast.. J Biol Chem.

[45] Jazwinski SM, Kim S, Dai J, Li L, Bi X (2010). HRAS1 and LASS1 with APOE are associated with human longevity and healthy aging.. Aging Cell.

[46] Mucke L, Masliah E, Johnson WB, Ruppe MD, Alford M (1994). Synaptotrophic effects of human amyloid beta protein precursors in the cortex of transgenic mice.. Brain Res.

[47] Zhao L, Longo-Guess C, Harris BS, Lee JW, Ackerman SL (2005). Protein accumulation and neurodegeneration in the woozy mutant mouse is caused by disruption of SIL1, a cochaperone of BiP.. Nat Genet.

[48] Zhao L, Rosales C, Seburn K, Ron D, Ackerman SL (2010). Alteration of the unfolded protein response modifies neurodegeneration in a mouse model of Marinesco-Sjogren syndrome.. Hum Mol Genet.

[49] Ackerman SL, Kozak LP, Przyborski SA, Rund LA, Boyer BB (1997). The mouse rostral cerebellar malformation gene encodes an UNC-5-like protein.. Nature.

[50] Bechtold LS, Sundberg J, Dawnalyn B (2000). Ultrastructural Evaluation of Mouse Mutations.. Systematic Approach to Evaluation of Mouse Mutations.

[51] Bielawski J, Pierce JS, Snider J, Rembiesa B, Szulc ZM (2009). Comprehensive quantitative analysis of bioactive sphingolipids by high-performance liquid chromatography-tandem mass spectrometry.. Methods Mol Biol.

[52] Sambrook J, Fritsch EF, Maniatis T (1989). Molecular Cloning: a laboratory manual.

[54] Kageyama-Yahara N, Riezman H (2006). Transmembrane topology of ceramide synthase in yeast.. Biochem J.

