# Supplementary figures and images for: A Deficiency of Ceramide Biosynthesis Causes Cerebellar Purkinje Cell Neurodegeneration and Lipofuscin Accumulation
Source: PLoS Genet. 2011 May 19;7(5):e1002063. doi: 10.1371/journal.pgen.1002063 (PMC3098191; doi:10.1371/journal.pgen.1002063)

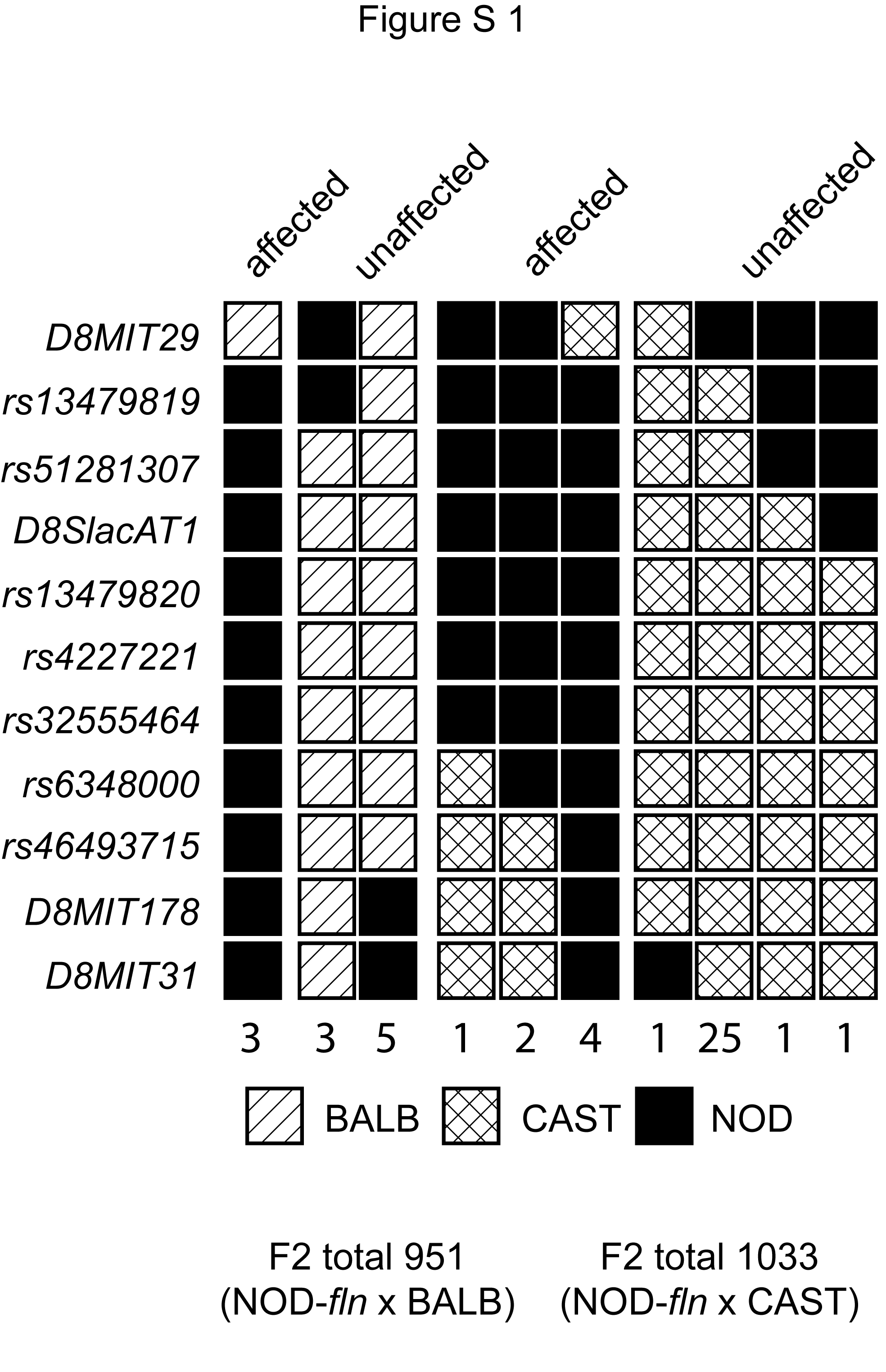

Supplement: Figure S1 — Haplotypes of selected recombinants used to refine the critical interval of the fln mutation. F2 recombinants from NOD.CB17-Prkdcscid/J-fln×BALB/cJ and NOD.CB17-Prkdcscid/J-fln×CAST/EiJ crosses are shown with the number of recombinants with each haplotype indicated. (TIF) [file pgen.1002063.s001.tif]

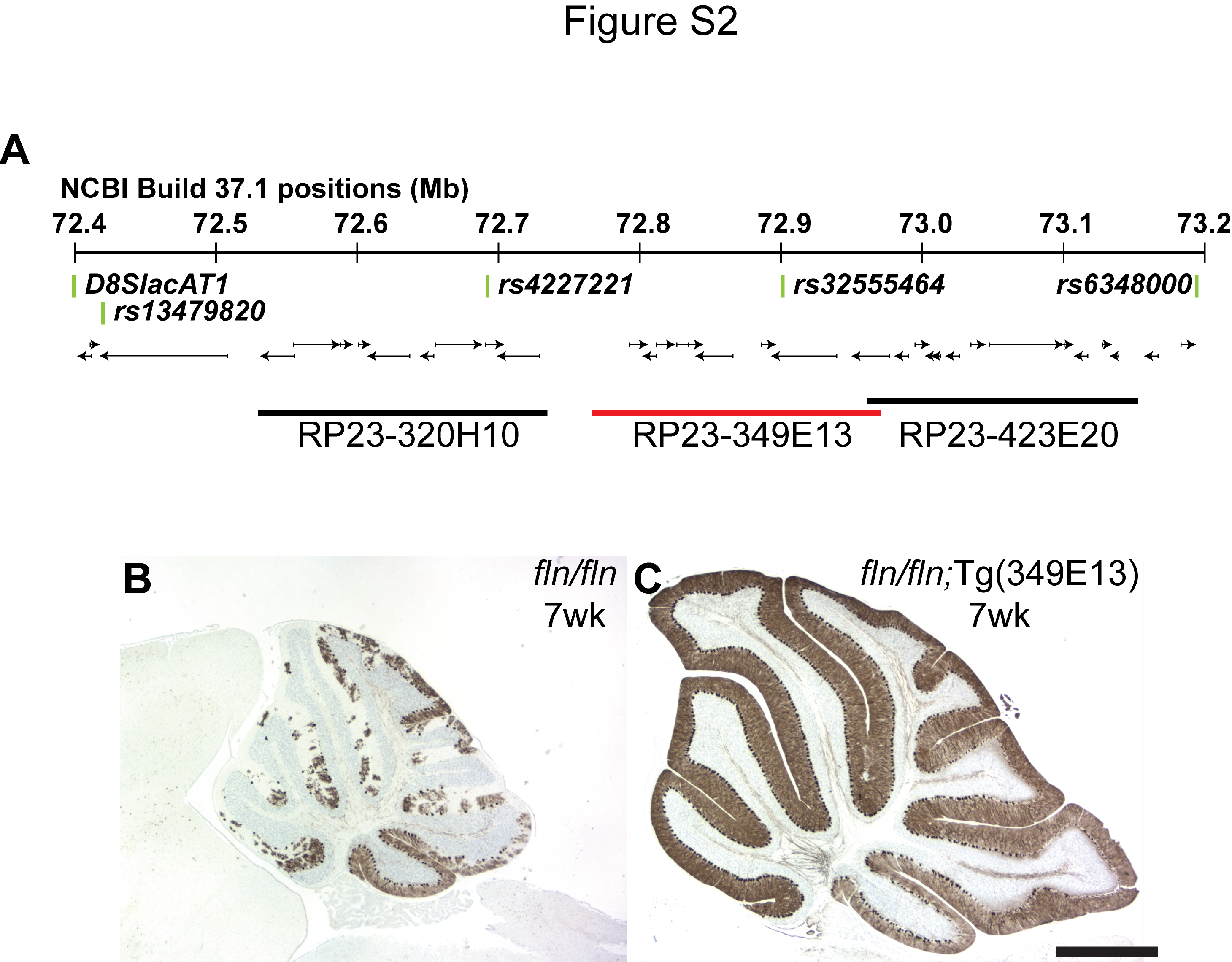

Supplement: Figure S2 — BAC rescue of the fln mutation. (A) The critical interval of the flincher mutation. The three BAC clones used in the BAC complementation experiments are shown. (B–C) Calbindin-D28 immunostaining of cerebellar sections from a seven-week-old fln/fln mouse and an age-matched fln/fln littermate carrying the BAC RP23-349E13 transgene. Scale bar: 1 mm (B–C). (TIF) [file pgen.1002063.s002.tif]

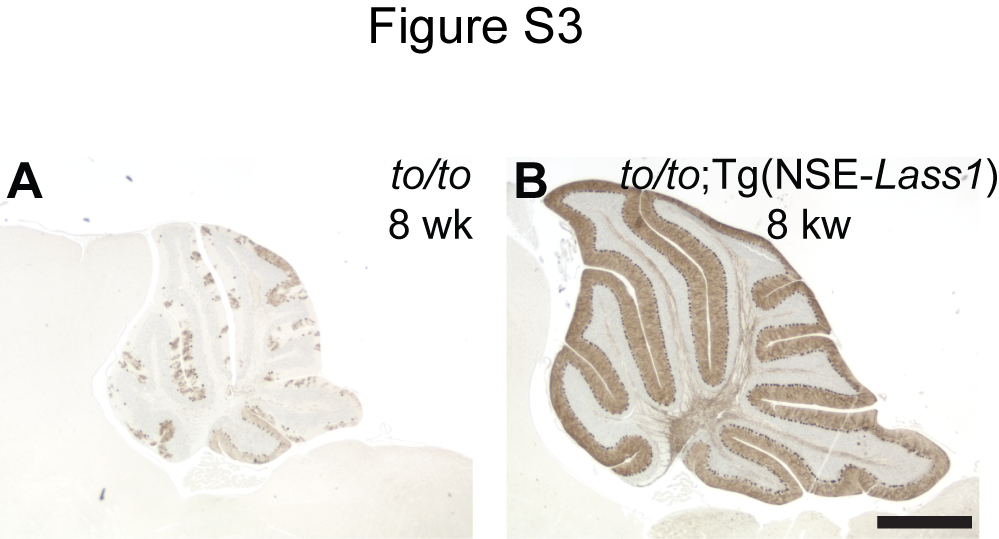

Supplement: Figure S3 — A neuron-specific Lass1 cDNA transgene suppresses the toppler mutation. Calbindin D-28 immunohistochemistry of cerebellar sections from eight-week-old to/to (A) and to/to; Tg(NSE-Lass1) (B) mice. Scale bar: 1 mm. (TIF) [file pgen.1002063.s003.tif]

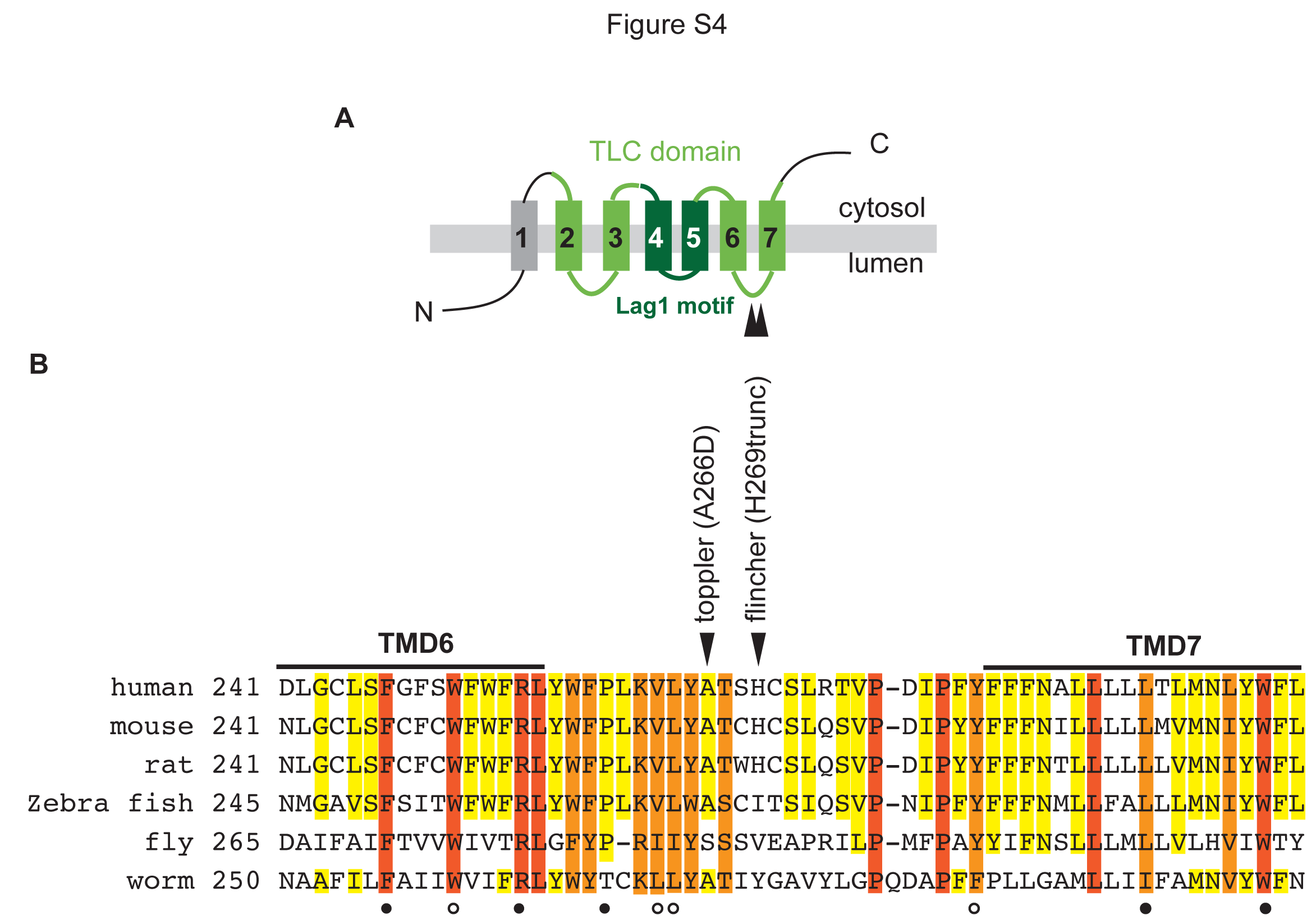

Supplement: Figure S4 — The fln and the to mutations are in the conserved TLC domain. (A) Predicted secondary structure of the mouse CerS1. Transmembrane domains were predicted with the TMPred server (http://www.ch.embnet.org/software/TMPRED_form.html) and combined with topological data of yeast ceramide synthases [53]. The to and the fln mutations are marked with arrowheads. The TLC domain is indicated in light green, and the LAG1 motif in the TLC domain is shaded in darker green. (B) Alignment of CerS1 orthologs using COBALT (http://www.ncbi.nlm.nih.gov/tools/cobalt/). Identical or similar residues across all species are colored red or orange, respectively. Residues conserved in vertebrates are indicated in yellow. Transmembrane domains were marked according to the mouse CerS1 sequence. Arrowheads denote the fln and the to mutations. Residues that are identical or conserved in mouse CerS1–CerS6 are marked with filled and open circles, respectively. Sequences used for analysis are NP_067090 (human), NP_619588 (mouse), NP_001037695 (rat), CAK11083 (Zebra fish), NP_727075 (Drosophila, fly), and NP493403 (C. elegans, worm). (TIF) [file pgen.1002063.s004.tif]

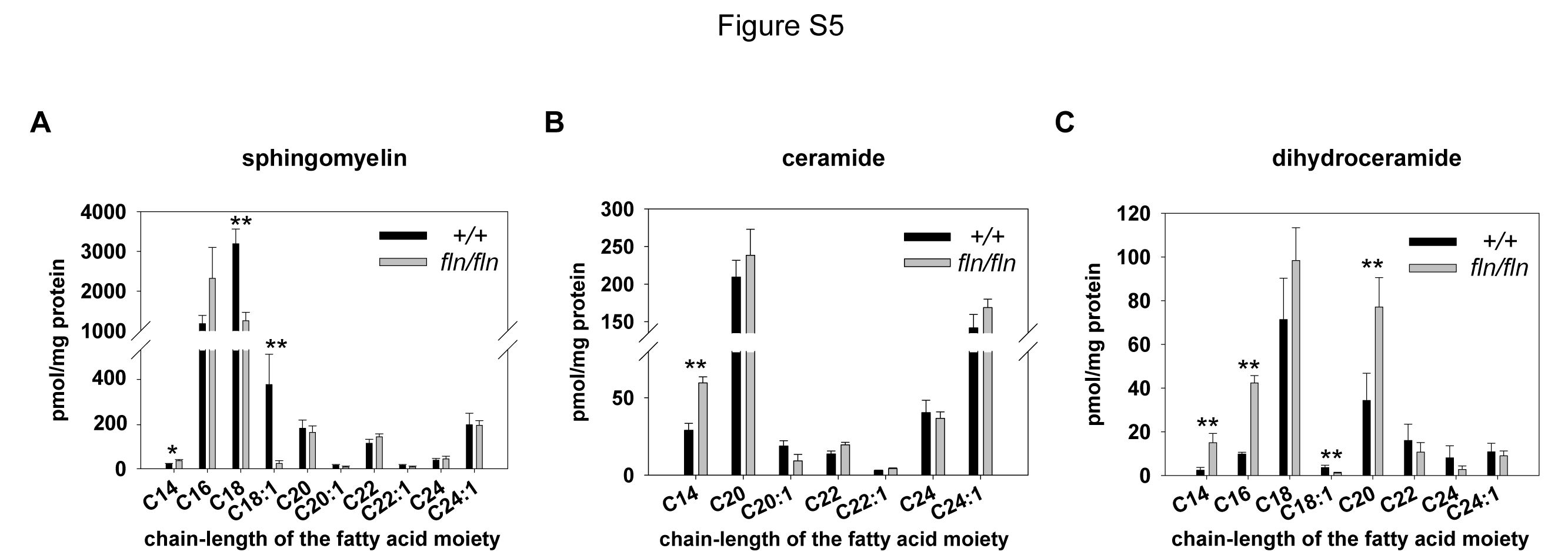

Supplement: Figure S5 — Mass spectrometry data showing profound changes of sphingolipids in the fln mutant brain. Brain sphingolipid levels in 12- to 13-day-old wild type (+/+, open bars; n = 5) and fln/fln mouse brains (filled bars; n = 6) were measured by mass spectrometry and normalized to protein concentration. (A) Abundance of sphingomyelin with different fatty acyl chain moieties. (B) Abundance of ceramide with different fatty acyl chain moieties. Note that C16 and C18 ceramide species are shown in Figure 4, and are not included here. (C) Abundance of dihydroceramide with different fatty acyl chain moieties. All values are mean ± SD. *: p≤0.05; **: p≤0.01. (TIF) [file pgen.1002063.s005.tif]
