# Supplementary material for: A Deficiency of Ceramide Biosynthesis Causes Cerebellar Purkinje Cell Neurodegeneration and Lipofuscin Accumulation
Source: PLoS Genet. 2011 May 19;7(5):e1002063. doi: 10.1371/journal.pgen.1002063 (PMC3098191; doi:10.1371/journal.pgen.1002063)
Supplement: Table S1 — Primers used to construct plasmids or used for genotyping. (DOC) [file pgen.1002063.s006.doc]

**Table S1.** Primers used to construct plasmids or used for genotyping.

| **Name** | **Sequence** |
| --- | --- |
| LZO319 | 5’-GGTTCAGTCGCTGTGACTCT-3’ |
| LZO320 | 5’-GAGGATCTGGTCATCCACTG-3’ |
| LZO324 | 5’-ACAGCAGCTGGTCTGACTTC-3’ |
| LZO327 | 5’-AATAAAGGCACAGTGTGGTC-3’ |
| LZO339 | 5’-CAAGGACATACAGCAACTCA-3’ |
| LZO396 | 5’-TGGTTCTTCTGAGCGTTCTACA-3’ |
| LZO426 | 5’-CGGAGTCAGTATCCCAGGTAGGA-3’ |
| LZO428 | 5’-AAACCCTGTGGCTGCCACA-3’ |
| LZO445 | 5’-GCTGGAGGCTGAGAGTGAAG-3’ |
| LZO446 | 5’-GAAGAAGAGCACGGAGATGG-3’ |
| LZO455 | 5’-CGCGGATCCTTGGATAGCATGGCTGCTGC-3’ |
| LZO456 | 5’-GATCGATCACTAGTCAAGAGACTCAGAAGAGCTTGTC-3’ |
| LZO463 | 5’-ATGGCAGTGCCCTGGGACAT-3’ |
| LZO477 | 5’-ACTTGCCACTGCAGCCTGCA-3’ |
| LZO478 | 5’-GGCGCTCAGCTGAATTCGAG-3’ |
| LZO479 | 5’-ATGGTAGGGAGGAGCTGGCA-3’ |
| LZO605 | 5’-CTGCCACTCCATCTATGCCA-3’ |
| LZO606 | 5’-CGAAAGCCACAATGTACAGGAAC-3’ |
| ROS BAC SP6 | 5’-GTTTTTTGCGATCTGCCGTTTC-3’ |
| BAC T7 | 5’-ACTCACAATACGACTCACTATAGGGAGAG-3’ |
| Lass1e5-P1 | 5’-GGCACAGACTGCAGGCTGCTT-3’ |
| Lass1e5-P2 | 5’-AGTACGGAATGTCACCCACAGACTGCAGGCTCCAG-3’ |
| Lass1e5-P3 | 5’-TGGTGAGGATGGTAGGGAGGAGCT-3’ |
| Lass1(toppler)e5-P1 | 5’-CTGCAGGCTGCAGTGGCAAGTCT-3’ |
| Lass1(toppler)e5-P2 | 5’-ATGTCAGGCACAGAGAGCAGGCTGCAGTGGCAACTGG-3’ |
