# Supplementary material for: A Deficiency of Ceramide Biosynthesis Causes Cerebellar Purkinje Cell Neurodegeneration and Lipofuscin Accumulation
Source: PLoS Genet. 2011 May 19;7(5):e1002063. doi: 10.1371/journal.pgen.1002063 (PMC3098191; doi:10.1371/journal.pgen.1002063)
Supplement: Text S1 — Genotyping assays. (DOC) [file pgen.1002063.s007.doc]

**Methods S1**

**Genotyping assays.** BAC transgenic lines generated with BAC RP23-349E13 were genotyped with primers ROS BAC SP6 and LZO320, and BAC T7 and LZO319. BAC transgenic lines generated with BAC RP23-320H10 were genotyped with primers ROS BAC SP6 and LZO327, and BAC T7 and LZO396. Primer sets BAC T7 and LZO339, and ROS BAC SP6 and LZO324, were used to genotype BAC transgenic lines generated with BAC RP23-423E20. *Lass1* cDNA transgenic line Tg(NSE*-Lass1*) was genotyped with primers LZO477 and LZO478. To genotype flincher and toppler alleles, exon 5 and flanking intron sequence of *Lass1* gene was amplified using LZO479 and LZO428, and followed by sequencing. Allele-specific PCR genotyping assays were also developed as described [Rust, et al]. To differentiate the flincher (*fln*) allele and the wild type allele, Lass1e5-P1 (corresponding to the wild type allele), Lass1e5-P2 (corresponding to the *fln* allele) and Lass1e5-P3 (the common primer for both alleles) were used. To differentiate the toppler (*to*) allele and the wild type allele, Lass1(toppler)e5-P1 (corresponding to the wild type allele), Lass1(toppler)e5-P2 (corresponding to the *to* allele) and Lass1e5-P3 (the common primer for both the wild type and the *to* alleles) were used.

Rust S, Funke H, Assmann G (1993) Mutagenically separated PCR (MS-PCR): a highly specific one step procedure for easy mutation detection. Nucleic Acids Res 21: 3623-3629.
